# Machine learning–driven risk prediction of delayed cerebral ischemia after aneurysmal subarachnoid hemorrhage using peripheral inflammatory markers

**DOI:** 10.3389/fneur.2025.1713341

**Published:** 2025-12-11

**Authors:** Yuanyuan Liu, Chengchen Li, Honglin Wang

**Affiliations:** 1 Chengdu Women's and Children's Central Hospital, School of Medicine, University of Electronic Science and Technology of China, Chengdu, China; 2 Department of Interventional Medicine, Dazhou Central Hospital, Dazhou, China

**Keywords:** machine learning, risk stratification, delayed cerebral ischemia (DCI), peripheral inflammatory biomarkers, aneurysmal subarachnoid hemorrhage (aSAH)

## Abstract

**Background:**

Delayed cerebral ischemia (DCI) remains a leading cause of secondary neurological deterioration and mortality after aneurysmal subarachnoid hemorrhage (aSAH). Accumulating evidence highlights the pivotal role of systemic inflammation in the pathogenesis of DCI, with peripheral inflammatory markers showing potential as early indicators. However, the predictive performance of individual biomarkers is limited. By leveraging machine learning (ML) techniques, it is possible to integrate heterogeneous inflammatory signals and model complex nonlinear relationships to improve individualized risk prediction.

**Methods and materials:**

We conducted a retrospective analysis of 562 aSAH patients admitted to a single tertiary center. Clinical, radiographic, and laboratory data—including peripheral inflammatory indices—were extracted from electronic medical records. The Boruta algorithm was applied for feature selection. Six ML models were developed and compared: logistic regression, neural network, random forest, support vector machine, gradient boosting machine (GBM), and extreme gradient boosting (XGBoost). Model performance was evaluated using the area under the receiver operating characteristic curve (AUC), sensitivity, specificity, F1 score, calibration curves, and decision curve analysis (DCA).

**Results:**

Among the six models, the neural network demonstrated the best balance between discrimination and calibration, with an AUC of 0.826 in the training cohort and 0.808 in the internal testing cohort. Eight predictors were included in the final model: Glasgow Coma Scale (GCS), Hunt-Hess grade, modified Fisher score, prognostic nutritional index (PNI), neutrophil-to-albumin ratio (NAR), neutrophil-to-lymphocyte platelet ratio (NLPR), C-reactive protein-to-lymphocyte ratio (CLR), and procalcitonin. SHapley Additive exPlanations (SHAP) analysis revealed Hunt-Hess grade and procalcitonin as top contributors.

**Conclusion:**

This study proposes a machine learning–based risk prediction tool for DCI after aSAH, built from routinely available inflammatory and clinical variables. The model demonstrated strong discriminative and calibration performance and provides a clinically interpretable, preoperative decision-support tool. Prospective multicenter validation is warranted to assess generalizability and facilitate clinical translation.

## 1 Introduction

Aneurysmal subarachnoid hemorrhage (aSAH) is a neurological emergency with high rates of mortality and long-term disability, accounting for a significant proportion of stroke-related morbidity (1). Despite advances in early management, more than 50% of survivors experience persistent neurological or cognitive deficits (2, 3). Delayed cerebral ischemia (DCI), occurring in 20–30% of patients between days 4–14 post-hemorrhage, is a major contributor to poor outcomes and increased healthcare burden (4–8). Its insidious onset and nonspecific symptoms hinder timely diagnosis and intervention.

There is an urgent clinical need for early, individualized risk prediction of DCI. Increasing evidence highlights the role of systemic inflammation in its pathogenesis (9–11). Blood–brain barrier disruption, microvascular dysfunction, and cytokine release after aSAH contribute to cerebral ischemia. Several peripheral inflammatory biomarkers—such as neutrophil × lymphocyte to platelet ratio (NLPR), procalcitonin (PCT), C-reactive protein-to-lymphocyte ratio (CLR), and systemic immune-inflammation index (SII)—have been explored for predicting DCI, yet single indices lack sufficient discriminative power.

Machine learning (ML) algorithms can address this limitation by modeling complex nonlinear interactions among multiple clinical and inflammatory variables (12–14). Unlike traditional statistical methods, ML enables data-driven feature selection and individualized risk estimation. Interpretability techniques such as Shapley Additive exPlanations (SHAP) further enhance transparency and support clinical integration.

In this study, we retrospectively analyzed 562 aSAH patients and developed multiple ML models to predict DCI using routinely collected inflammatory and clinical parameters. We applied Boruta for feature selection, compared six algorithms, and assessed performance in terms of discrimination, calibration, and clinical utility. Our goal was to build an interpretable, EMR-compatible risk prediction tool to support early stratification and decision-making in aSAH.

## 2 Materials and methods

### 2.1 Study design and population

This retrospective cohort study was conducted at a tertiary medical center and designed according to the Declaration of Helsinki and the STROBE guidelines. A total of 562 patients diagnosed with aSAH were included. Diagnosis was confirmed via cranial CT, CTA, or DSA, based on criteria from the Clinical Practice Guidelines for SAH.

Inclusion criteria were: (1) spontaneous SAH; (2) admission within 24 h of symptom onset; (3) blood tests and CT performed within 24 h of admission; (4) aneurysm secured by surgical clipping or endovascular coiling within 72 h; and (5) DCI occurring between days 4–14 after aSAH.

Exclusion criteria included: (1) SAH from trauma, AVM, AVF, or other non-aneurysmal causes; (2) recurrent aSAH or reoperation; (3) in-hospital death; (4) comorbid infection, autoimmune disease, malignancy, uremia, cirrhosis, chronic heart/pulmonary disease; or (5) history of stroke with residual deficits.

### 2.2 Data collection

Demographic and clinical data were extracted from electronic medical records, including age, sex, smoking and alcohol history, hypertension, diabetes mellitus, coronary artery disease, anticoagulant use, and other comorbidities. Neurological status on admission was assessed using the World Federation of Neurosurgical Societies (WFNS) grade, Hunt–Hess grade, and modified Fisher score. To evaluate systemic inflammation and immune-nutritional status, several derived indices were calculated from routine laboratory tests, including the prognostic nutritional index (PNI = albumin + 5 × lymphocyte count), neutrophil-to-albumin ratio (NAR), platelet-to-albumin ratio (PAR), neutrophil × lymphocyte to platelet ratio (NLPR), monocyte-to-Lymphocyte Ratio (MLR), systemic Inflammation Response Index (SIRI) and platelet-to-lymphocyte ratio (PLR). These indices have been recognized in previous studies as surrogate markers of inflammatory activation and immunonutritional imbalance in critical illness. Importantly, all laboratory parameters used for these calculations were obtained from the first routine blood test performed within 24 h after admission, typically collected by trained nurses, to ensure data consistency and minimize variability related to timing. During hospitalization, all patients received standardized treatments in accordance with established management guidelines for aSAH, including blood pressure control, intracranial pressure reduction, and prevention of cerebral vasospasm (CVS).

### 2.3 Outcome definition

DCI was defined according to the international consensus criteria proposed by Vergouwen et al. (15), supplemented by current clinical practice. DCI was considered present if it occurred between days 4 and 14 following aSAH and was not attributable to other causes such as rebleeding, infection, seizures, or metabolic disturbances. The diagnosis required at least one of the following: (1) new focal neurological deficits, including hemiparesis, aphasia, hemianopia, or neglect, lasting more than 1 h; (2) a decrease of two or more points in the Glasgow Coma Scale (GCS) score, either in individual domains or total score, sustained for at least 1 h; (3) new ischemic lesions on CT or MRI not observed on initial or early postoperative imaging; or (4) in patients with impaired consciousness, DCI was inferred from ancillary findings such as regional hypoperfusion on CT perfusion imaging (CTP), elevated cerebral blood flow velocity on transcranial Doppler (TCD), or cortical dysfunction indicated by diffuse slow-wave activity on electroencephalography (EEG). All cases were reviewed and confirmed by experienced neurosurgeons and neuroradiologists through clinical and imaging consensus.

### 2.4 Statistical analysis

All analyses were performed using SPSS version 27.0 (IBM Corp., Armonk, NY, USA), GraphPad Prism version 10.1.2 (GraphPad Software, San Diego, CA, USA), and RStudio version 4.4.2. Feature selection was conducted using the Boruta algorithm, a random forest–based wrapper method that introduces shadow features to identify variables with significant predictive importance. Based on the selected features, six supervised machine learning models were developed: logistic regression, neural network, random forest (RF), support vector machine (SVM), gradient boosting machine (GBM), and extreme gradient boosting (XGBoost).

During model development, all hyperparameters were systematically tuned using grid-search optimization, and 10-fold cross-validation was applied to reduce the risk of overfitting and enhance model stability. The key hyperparameter settings, tuning ranges, and final optimal values for each model are provided in Supplementary File S1 for transparency and reproducibility.

Model performance was evaluated using accuracy, area under the receiver operating characteristic curve (AUC), sensitivity, specificity, and F1 score. ROC curves and AUC values were computed using the “pROC” package. Calibration was assessed with Brier scores and calibration curves generated using the “caret” package, where lower Brier scores indicated better agreement between predicted probabilities and observed outcomes. Decision curve analysis (DCA) was performed using the “ggDCA” package to evaluate clinical utility across different threshold probabilities. To enhance interpretability, Shapley Additive explanations (SHAP) were implemented using the “shapviz” package to visualize feature contributions at both global and individual levels. Additional R packages used for data preprocessing, modeling, and visualization included tidyverse, ggplot2, rms, and rmda.

## 3 Results

### 3.1 Participants characteristics

A total of 562 patients with aSAH were included and randomly assigned to a training cohort (*n* = 393) and a testing cohort (*n* = 169). Baseline characteristics were largely comparable between the two groups, as shown in Table 1. The median age of the overall cohort was 58.0 years (IQR: 51.0–66.8), with no significant difference between cohorts (*p* = 0.506). Inflammatory and nutritional indicators—including albumin, fibrinogen, PNI, NAR, PAR, NLPR, MLR, SIRI, CLR, procalcitonin, CRP, blood glucose, and creatinine—were similarly distributed (*p* > 0.05), except for creatinine, which showed a borderline difference (*p* = 0.072). Categorical variables such as sex, smoking, alcohol use, hypertension, Hunt–Hess grade, modified Fisher score, aneurysm location, surgical modality, and GCS score were also balanced between groups (*p* > 0.05). The only significant difference was the prevalence of diabetes mellitus, which was higher in the testing cohort (10.65%) than in the training cohort (5.09%) (*p* = 0.016).

**Table 1:** Baseline characteristics of the study population across training, internal test, and external validation cohorts.

Variables	Total (*n* = 562)	Test (*n* = 169)	Train (*n* = 393)	*p*
Age, M (Q₁, Q₃)	58.00 (51.00, 66.75)	57.00 (50.00, 66.00)	58.00 (51.00, 67.00)	0.506
Albumin, M (Q₁, Q₃)	38.90 (34.60, 42.30)	38.40 (34.10, 42.30)	39.10 (34.60, 42.30)	0.770
Fibrinogen, M (Q₁, Q₃)	3.00 (2.50, 3.68)	3.00 (2.40, 3.70)	3.00 (2.50, 3.60)	0.959
PNI, M (Q₁, Q₃)	44.67 (39.88, 48.83)	44.70 (40.05, 48.20)	44.65 (39.85, 49.15)	0.638
NAR, M (Q₁, Q₃)	0.24 (0.18, 0.31)	0.22 (0.17, 0.30)	0.25 (0.18, 0.31)	0.104
PAR, M (Q₁, Q₃)	5.01 (4.06, 6.22)	4.95 (4.00, 6.26)	5.02 (4.08, 6.19)	0.815
NLPR, M (Q₁, Q₃)	0.05 (0.03, 0.08)	0.04 (0.03, 0.08)	0.05 (0.03, 0.08)	0.684
MLR, M (Q₁, Q₃)	0.53 (0.33, 0.89)	0.48 (0.32, 0.92)	0.55 (0.35, 0.88)	0.369
SIRI, M (Q₁, Q₃)	5.04 (2.60, 9.69)	4.24 (2.23, 9.05)	5.20 (2.81, 9.80)	0.139
CLR, M (Q₁, Q₃)	6.07 (1.64, 20.08)	6.30 (1.75, 24.78)	5.91 (1.60, 19.29)	0.495
Procalcitonin, M (Q₁, Q₃)	0.14 (0.05, 0.60)	0.15 (0.06, 0.66)	0.14 (0.05, 0.58)	0.596
CRP, M (Q₁, Q₃)	5.90 (1.72, 19.90)	6.40 (1.90, 19.60)	5.60 (1.70, 20.10)	0.511
Sugar, M (Q₁, Q₃)	7.60 (6.30, 9.10)	7.40 (6.20, 9.60)	7.60 (6.40, 8.90)	0.840
Creatinine, M (Q₁, Q₃)	54.50 (45.00, 67.00)	56.00 (46.00, 69.00)	53.00 (45.00, 66.00)	0.072
DCI, *n* (%)				0.625
No	384 (68.33)	113 (66.86)	271 (68.96)	
Yes	178 (31.67)	56 (33.14)	122 (31.04)	
Sex, *n* (%)				0.212
Male	224 (39.86)	74 (43.79)	150 (38.17)	
Female	338 (60.14)	95 (56.21)	243 (61.83)	
Smoke, *n* (%)				0.428
No	413 (73.49)	128 (75.74)	285 (72.52)	
Yes	149 (26.51)	41 (24.26)	108 (27.48)	
Drink, *n* (%)				0.296
No	440 (78.29)	137 (81.07)	303 (77.10)	
Yes	122 (21.71)	32 (18.93)	90 (22.90)	
Hypertension, *n* (%)				0.899
No	265 (47.15)	79 (46.75)	186 (47.33)	
Yes	297 (52.85)	90 (53.25)	207 (52.67)	
Diabetes, *n* (%)				**0.016**
No	524 (93.24)	151 (89.35)	373 (94.91)	
Yes	38 (6.76)	18 (10.65)	20 (5.09)	
GCS, *n* (%)				0.287
13–15	300 (53.38)	84 (49.70)	216 (54.96)	
8–12	216 (38.43)	67 (39.64)	149 (37.91)	
3–7	46 (8.19)	18 (10.65)	28 (7.12)	
Hunt–Hess, *n* (%)				0.197
I, II	342 (60.85)	96 (56.80)	246 (62.60)	
III, IV, V	220 (39.15)	73 (43.20)	147 (37.40)	
Modified fisher, *n* (%)				0.645
I, II	331 (58.90)	102 (60.36)	229 (58.27)	
III, IV	231 (41.10)	67 (39.64)	164 (41.73)	
Aneurysm location, *n* (%)				0.676
Anterior circulation	532 (94.66)	161 (95.27)	371 (94.40)	
Posterior circulation	30 (5.34)	8 (4.73)	22 (5.60)	
Surgical method, *n* (%)				0.376
Endovascular treatment	397 (70.64)	115 (68.05)	282 (71.76)	
Surgical clipping	165 (29.36)	54 (31.95)	111 (28.24)	

DCI, Delayed cerebral ischemia; GCS, Glasgow Coma Scale; WFNS, World Federation of Neurosurgical Societies; PNI, prognostic nutritional index; NAR, neutrophil-to-albumin ratio; NLPR, neutrophil/(lymphocyte × platelet) ratio; MLR, Monocyte-to-Lymphocyte Ratio; SIRI, Systemic Inflammation Response Index; CLR, C-reactive protein-to-lymphocyte ratio; PAR, Platelet-to-Albumin Ratio.

Data are presented as *n* (%). Variables were grouped according to clinical criteria or quartile distribution. *p* values were calculated using Pearson’s chi-square test or Fisher’s exact test for categorical variables. A two-sided *p* < 0.05 was considered statistically significant. Bold values represent *p* < 0.05, considered statistically significant.

In the training cohort, DCI occurred in 122 patients (31.04%). Comparisons between the DCI and non-DCI groups are summarized in Table 2. Patients who developed DCI exhibited significantly elevated levels of NAR (0.28 vs. 0.23, *p* < 0.001), MLR (0.62 vs. 0.51, *p* = 0.007), SIRI (7.69 vs. 4.65, *p* < 0.001), procalcitonin (0.30 vs. 0.11 ng/mL, *p* < 0.001), and blood glucose (7.80 vs. 7.40 mmol/L, *p* = 0.040). No significant differences were found for age, albumin, PNI, NLPR, CLR, or creatinine (*p* > 0.05). Additionally, patients with DCI were more likely to present with lower GCS scores (*p* < 0.001), higher Hunt–Hess grades, and higher modified Fisher scores (both *p* < 0.001). The distribution of surgical modalities also differed significantly between groups (*p* < 0.05).

**Table 2:** Demographic characteristics of patients with different outcomes in the training cohort.

Variables	Total (*n* = 393)	No-DCI (*n* = 271)	DCI (*n* = 122)	*p*
Age, M (Q₁, Q₃)	58.00 (51.00, 67.00)	58.00 (51.00, 66.00)	59.00 (52.00, 68.00)	0.264
Albumin, M (Q₁, Q₃)	39.10 (34.60, 42.30)	39.10 (34.70, 42.30)	39.05 (34.70, 42.58)	0.889
Fibrinogen, M (Q₁, Q₃)	3.00 (2.50, 3.60)	3.00 (2.50, 3.70)	3.00 (2.42, 3.60)	0.539
PNI, M (Q₁, Q₃)	44.65 (39.85, 49.15)	44.55 (39.98, 49.08)	45.02 (39.75, 49.19)	0.801
NAR, M (Q₁, Q₃)	0.25 (0.18, 0.31)	0.23 (0.18, 0.30)	0.28 (0.20, 0.35)	**<0.001**
PAR, M (Q₁, Q₃)	5.02 (4.08, 6.19)	5.02 (4.08, 6.23)	5.02 (4.10, 6.17)	0.958
NLPR, M (Q₁, Q₃)	0.05 (0.03, 0.08)	0.05 (0.03, 0.07)	0.06 (0.03, 0.10)	0.144
MLR, M (Q₁, Q₃)	0.55 (0.35, 0.88)	0.51 (0.34, 0.82)	0.62 (0.36, 1.12)	**0.007**
SIRI, M (Q₁, Q₃)	5.20 (2.81, 9.80)	4.65 (2.60, 8.71)	7.69 (3.25, 12.69)	**<0.001**
CLR, M (Q₁, Q₃)	5.91 (1.60, 19.29)	6.07 (1.61, 16.96)	5.57 (1.50, 27.14)	0.447
Procalcitonin, M (Q₁, Q₃)	0.14 (0.05, 0.58)	0.11 (0.04, 0.40)	0.30 (0.07, 1.10)	**<0.001**
CRP, M (Q₁, Q₃)	5.60 (1.70, 20.10)	5.60 (1.75, 17.25)	5.50 (1.42, 28.05)	0.447
Sugar, M (Q₁, Q₃)	7.60 (6.40, 8.90)	7.40 (6.20, 8.80)	7.80 (6.60, 9.40)	**0.040**
Creatinine, M (Q₁, Q₃)	53.00 (45.00, 66.00)	53.00 (45.00, 64.50)	54.00 (46.00, 68.00)	0.443
Sex, *n* (%)				0.223
Male	150 (38.17)	98 (36.16)	52 (42.62)	
Female	243 (61.83)	173 (63.84)	70 (57.38)	
Smoke, *n* (%)				0.709
No	285 (72.52)	195 (71.96)	90 (73.77)	
Yes	108 (27.48)	76 (28.04)	32 (26.23)	
Drink, *n* (%)				0.292
No	303 (77.10)	213 (78.60)	90 (73.77)	
Yes	90 (22.90)	58 (21.40)	32 (26.23)	
Hypertension, *n* (%)				0.210
No	186 (47.33)	134 (49.45)	52 (42.62)	
Yes	207 (52.67)	137 (50.55)	70 (57.38)	
Diabetes, *n* (%)				0.166
No	373 (94.91)	260 (95.94)	113 (92.62)	
Yes	20 (5.09)	11 (4.06)	9 (7.38)	
GCS, *n* (%)				**<0.001**
13–15	216 (54.96)	186 (68.63)	30 (24.59)	
8–12	149 (37.91)	83 (30.63)	66 (54.10)	
3–7	28 (7.12)	2 (0.74)	26 (21.31)	
Hunt–Hess, *n* (%)				**<0.001**
I, II	246 (62.60)	211 (77.86)	35 (28.69)	
III, IV, V	147 (37.40)	60 (22.14)	87 (71.31)	
Modified fisher, *n* (%)				**<0.001**
I, II	229 (58.27)	181 (66.79)	48 (39.34)	
III, IV	164 (41.73)	90 (33.21)	74 (60.66)	
Aneurysm location, *n* (%)				0.579
Anterior circulation	371 (94.40)	257 (94.83)	114 (93.44)	
Posterior circulation	22 (5.60)	14 (5.17)	8 (6.56)	
Surgical method, *n* (%)				**<0.001**
Endovascular treatment	282 (71.76)	209 (77.12)	73 (59.84)	
Surgical clipping	111 (28.24)	62 (22.88)	49 (40.16)	

DCI, Delayed cerebral ischemia; GCS, Glasgow Coma Scale; WFNS, World Federation of Neurosurgical Societies; PNI, prognostic nutritional index; NAR, neutrophil-to-albumin ratio; NLPR, neutrophil/(lymphocyte × platelet) ratio; MLR, Monocyte-to-Lymphocyte Ratio; SIRI, Systemic Inflammation Response Index; CLR, C-reactive protein-to-lymphocyte ratio; PAR, Platelet-to-Albumin Ratio.

Data are presented as *n* (%). Variables were grouped according to clinical criteria or quartile distribution. *p* values were calculated using Pearson’s chi-square test or Fisher’s exact test for categorical variables. A two-sided *p* < 0.05 was considered statistically significant. Bold values represent *p* < 0.05, considered statistically significant.

### 3.2 Feature selection

**Figure 1:**
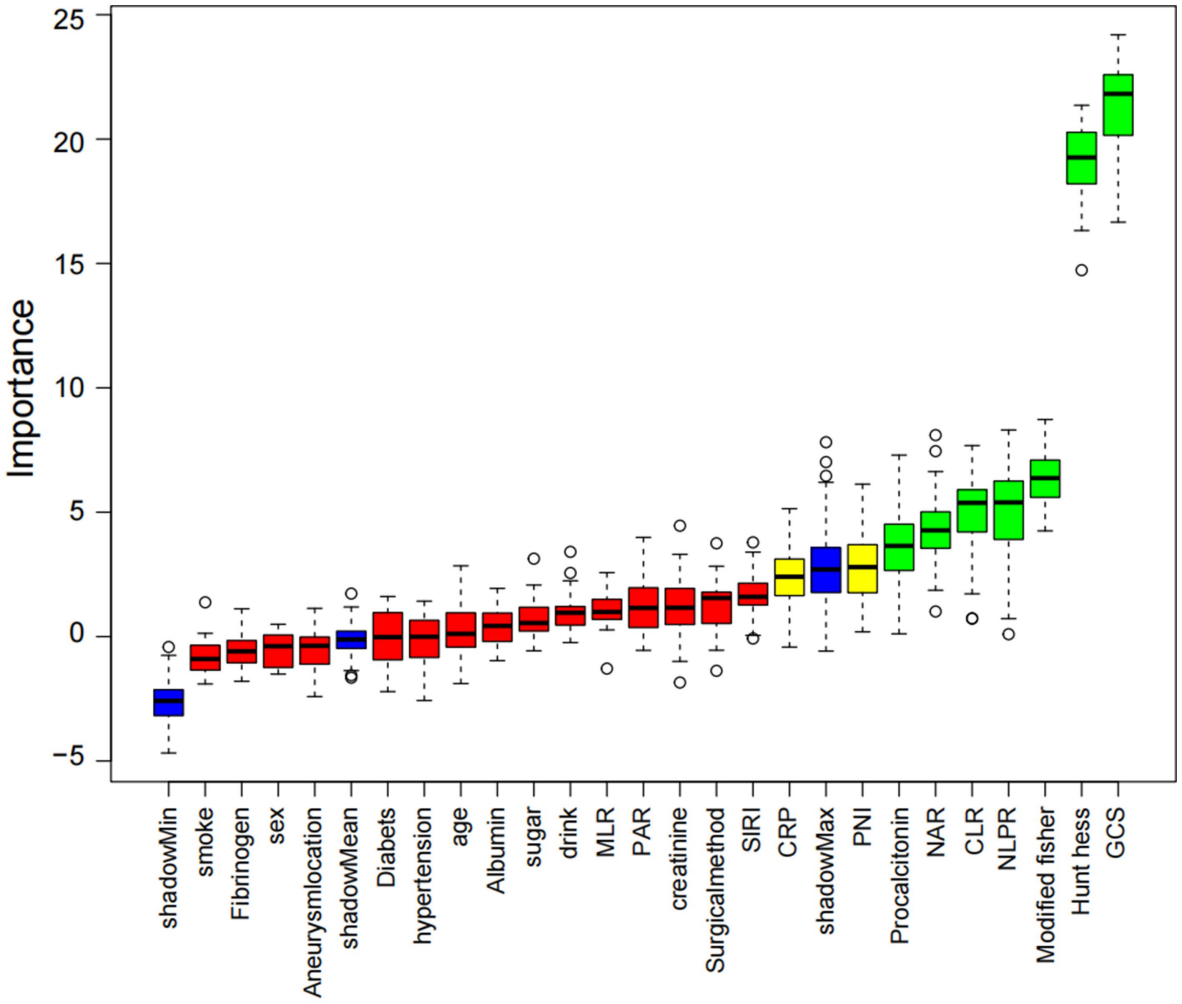
Feature selection process and final predictor selection. Feature importance identified by the Boruta algorithm (green: confirmed, red: rejected, yellow: tentative).

A total of 24 candidate predictors were initially included, encompassing clinical grading scales, radiological scores, and a range of inflammatory and nutritional biomarkers. To identify the most relevant variables associated with DCI, we applied the Boruta algorithm, a random forest–based wrapper method that introduces shuffled shadow features to assess the relative importance of original variables (Figure 1). After iterative comparisons and statistical filtering, eight variables were retained as core predictors: GCS, modified Fisher score, Hunt–Hess grade, PNI, NAR, NLPR, CLR, and procalcitonin (Figure 1). These features demonstrated high selection stability, strong discriminative potential, and good clinical interpretability, and were used for subsequent model construction.

**Figure 2:**
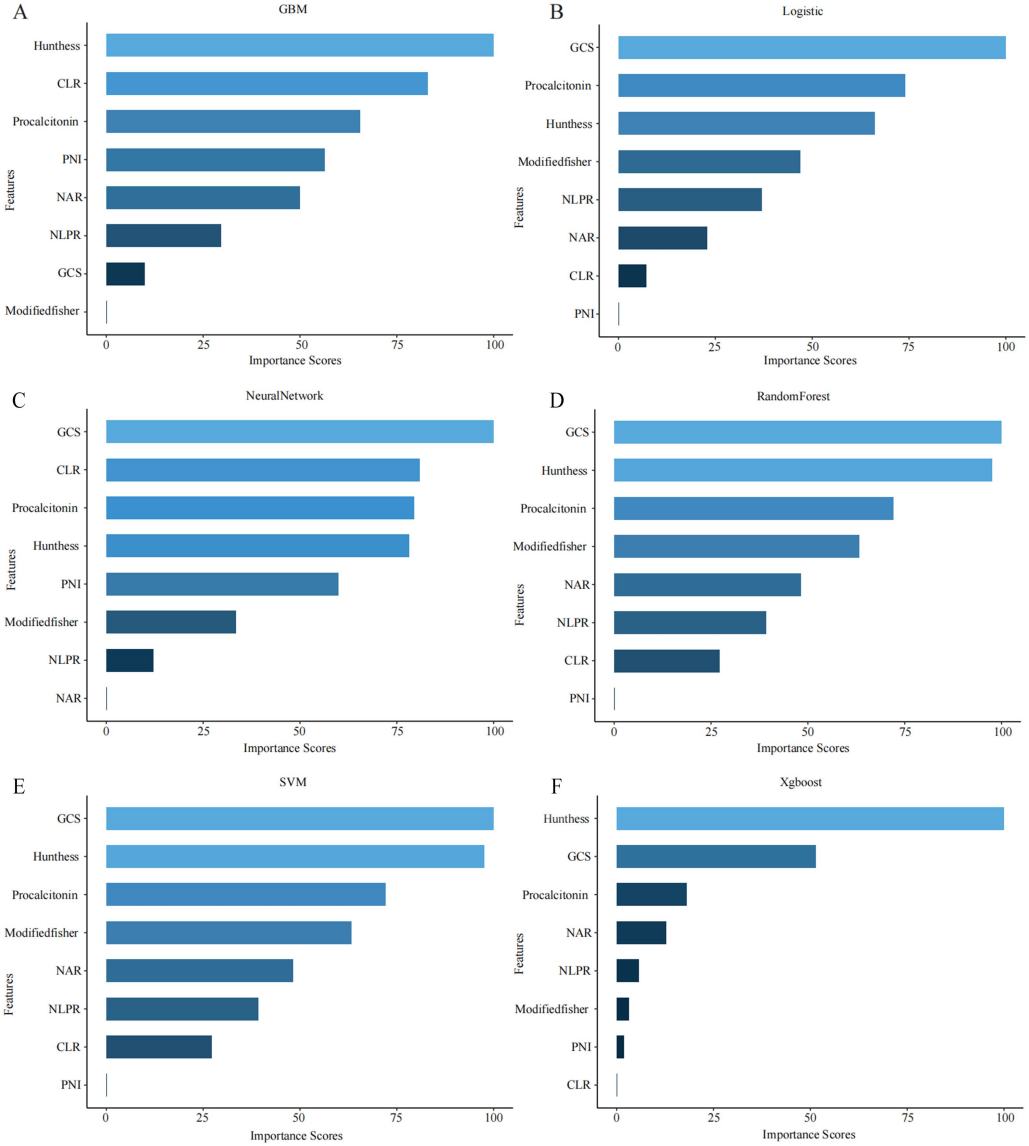
Top-ranked predictors across different machine learning models. **(A–F)** The figure shows the top 10 most important features identified by six machine learning algorithms: **(A)** GBM, **(B)** logistic regression (LR), **(C)** neural network (NN), **(D)** random forest (RF), **(E)** support vector machine (SVM), and **(F)** XGBoost. Feature importance was calculated using internal metrics specific to each model (e.g., standardized coefficients for LR, Gini importance for RF).

Subsequently, model-specific interpretability tools—including integrated gradients and permutation-based importance (in the neural network)—were used to assess and rank feature importance across all six machine learning algorithms (Figure 2). GCS score, CLR, and procalcitonin consistently ranked among the top predictors in multiple models, underscoring their pivotal role in the early identification of DCI risk.

### 3.3 Performance of machine learning models

**Figure 3:**
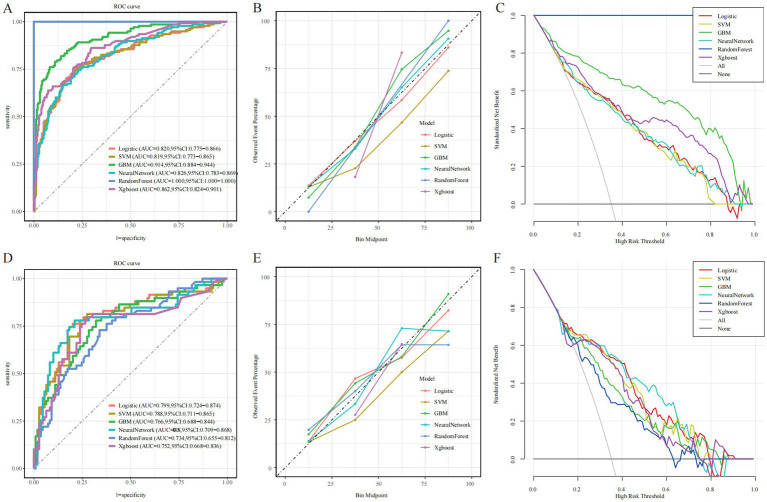
Evaluation of the predictive performance of six machine learning models. **(A,D)** Receiver operating characteristic (ROC) curves. **(B,E)** Calibration plots. **(C,F)** Decision curve analysis (DCA). Left, middle, and right columns represent the training, internal test, and external validation cohorts, respectively.

In the training cohort, the RF model demonstrated the best apparent performance in both discrimination and calibration, with an AUC of 1.00 (95% CI: 1.00–1.00), a Brier score of 0.00, and nearly perfect accuracy, sensitivity, and specificity (Figure 3A). However, such near-perfect performance strongly suggests overfitting, where the model may rely excessively on sample-specific patterns, limiting its generalizability to unseen data (Figure 3D). In comparison, the GBM model achieved a slightly lower training AUC of 0.914 (95% CI: 0.884–0.944) but still maintained stable performance across multiple metrics. While both RF and GBM performed well on training data, their AUCs declined markedly in the testing cohort, indicating a risk of overfitting and highlighting the need for cautious evaluation before clinical application. Notably, the neural network model achieved an AUC of 0.826 in the training cohort and 0.808 in the testing cohort, with a Brier score of 0.163 (Table 3). Although its training performance was lower than that of RF and GBM, the neural network exhibited better generalization with minimal performance drop, suggesting stronger adaptability to unseen data. These findings indicate that despite a slightly weaker initial fit, the neural network holds greater promise for real-world clinical use. In addition, DCA revealed that all six models yielded a positive net clinical benefit across a wide range of risk thresholds (0–0.8), further supporting their potential clinical utility (Figures 3C,F).

**Table 3:** Performance of six machine learning models in the training and test sets.

Data set	Model	Accuracy	Sensitivity	Specificity	Precision	F1 Score	Brier	C index
Train	LR	0.777	0.768	0.781	0.654	0.707	0.155	0.82
	SVM	0.772	0.746	0.785	0.652	0.696	0.167	0.819
	GBM	0.855	0.79	0.891	0.796	0.793	0.111	0.914
	Neural Network	0.761	0.746	0.77	0.636	0.687	0.157	0.826
	RF	1	1	1	1	1	0	1
	XGBoost	0.759	0.862	0.703	0.61	0.715	0.248	0.862
Test	LR	0.399	0.792	0.746	0.817	0.688	0.163	0.799
	SVM	0.412	0.768	0.78	0.761	0.639	0.176	0.788
	GBM	0.216	0.714	0.78	0.679	0.568	0.184	0.766
	Neural Network	0.344	0.806	0.78	0.837	0.687	0.163	0.808
	RF	0.21	0.685	0.729	0.661	0.537	0.207	0.734
	XGBoost	0.498	0.75	0.797	0.725	0.61	0.249	0.752

LR, logistic regression; SVM, support vector machine; GBM, gradient boosting machine; NN, neural network; RF, random forest; AUC, area under the curve.

**Figure 4:**
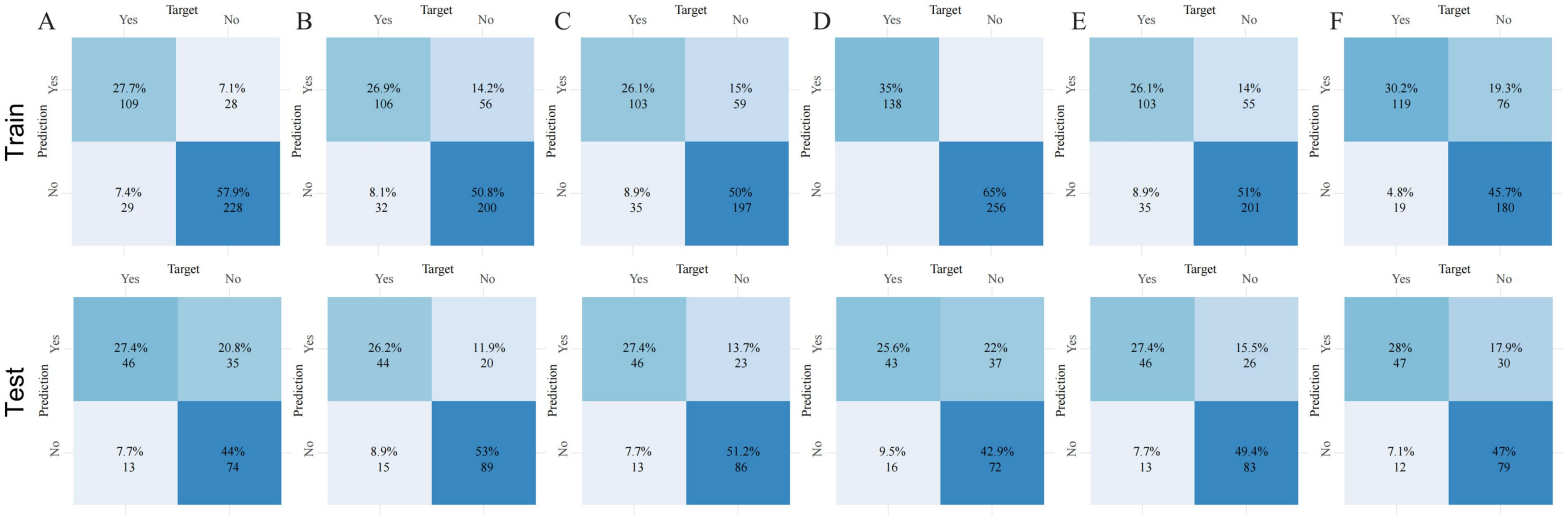
Confusion matrices of six machine learning models across three datasets. Panels **(A–F)** correspond to the following models: **(A)** gradient boosting machine (GBM), **(B)** logistic regression (LR), **(C)** neural network (NN), **(D)** random forest (RF), **(E)** extreme gradient boosting (XGBoost), and **(F)** support vector machine (SVM). Each panel shows the number of true positives (TP), false positives (FP), true negatives (TN), and false negatives (FN), enabling direct comparison of classification accuracy and error rates across models and datasets.

To evaluate calibration consistency across datasets, calibration curves were plotted for all six models, comparing predicted probabilities with observed event rates. In the training cohort, most models—especially RF and GBM—demonstrated systematic overestimation in the medium-to-high risk range (bin midpoint ≥ 50%), deviating from the ideal calibration line and indicating overfitting (Figure 3B). In contrast, the neural network showed closer alignment with the ideal curve in the low-to-medium risk range. In the testing cohort, overall calibration declined. RF and GBM showed pronounced deviations, overestimating event probabilities in the high-risk range, further confirming poor generalization (Figure 3E). By comparison, the neural network and logistic regression models maintained better calibration across the entire risk spectrum, suggesting more stable and reliable prediction. Confusion matrix analysis further supported the neural network’s generalization capability, showing a high proportion of true positives and true negatives and a relatively low misclassification rate in the testing cohort (Figure 4C). In contrast, the RF model exhibited a higher false-positive rate, particularly in the test set data, reflecting its weaker generalization performance (Figure 4D). The confusion matrices for the other models, including GBM (Figure 4A), logistic regression (Figure 4B), XGBoost (Figure 4E), and SVM (Figure 4F), show generally consistent performance, but they did not exhibit the same degree of overfitting seen in the RF model.

Considering overall discrimination, calibration, stability, and clinical net benefit, the neural network can be preliminarily regarded as the optimal model within the current dataset. Nonetheless, external validation remains essential to confirm its robustness and applicability in broader clinical settings.

### 3.4 Model interpretation

**Figure 5:**
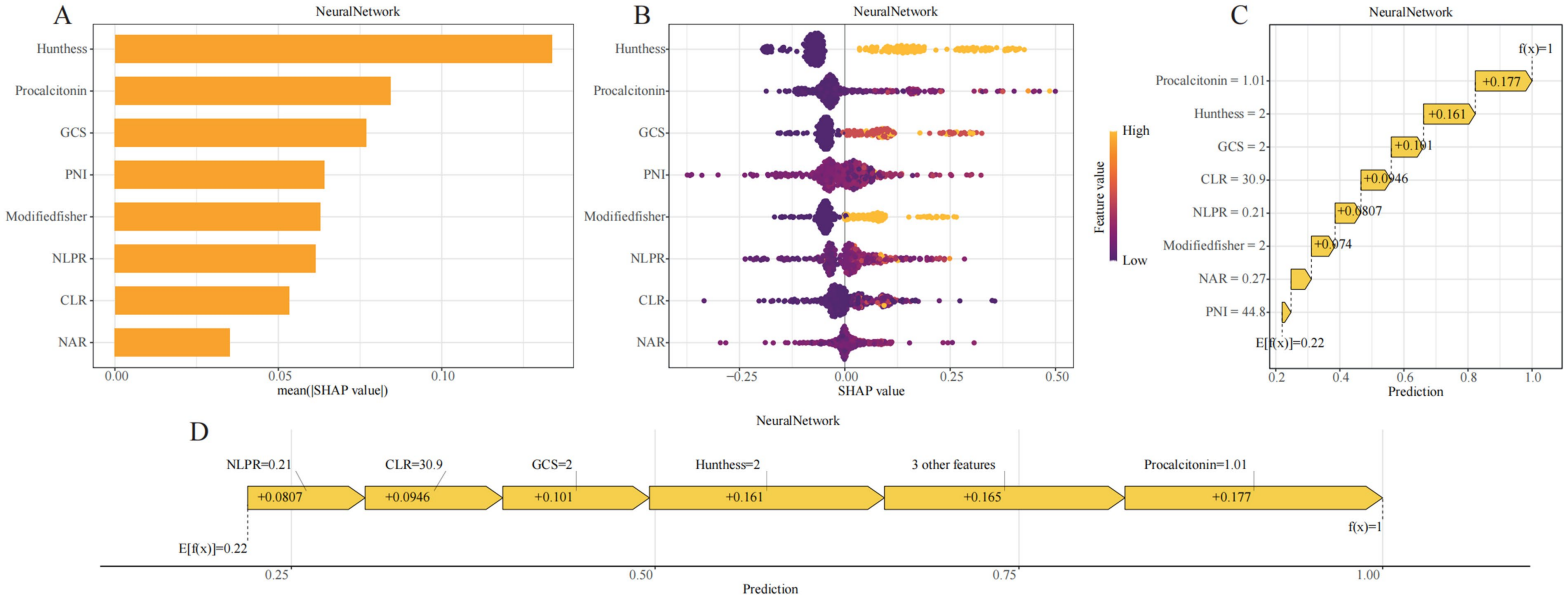
SHAP-based interpretability visualizations for the GBM model. **(A)** SHAP bar plot showing the mean absolute SHAP values across all samples, representing global feature importance. **(B)** SHAP beeswarm plot displaying the distribution of SHAP values for each feature across individual samples; color denotes the original feature value and direction of effect. **(C)** SHAP waterfall plot illustrating how the prediction for a representative patient evolves from the base value (E[f(x)]) to the final output (f(x)) through additive contributions of each variable. **(D)** SHAP force plot visualizing the positive and negative contributions of features in the same sample; arrow lengths represent effect magnitudes.

To enhance model transparency and support clinical interpretability, SHAP were applied to the neural network model. Global feature importance analysis revealed that Hunt–Hess grade, procalcitonin (PCT), GCS score, PNI, modified Fisher score, NLPR, CLR, and NAR were the top contributors to model predictions (Figure 5A). The SHAP summary plot illustrated both the magnitude and direction of each feature’s impact, showing that higher inflammatory markers and worse clinical grading scores were consistently associated with increased DCI risk (Figure 5B).

At the individual level, SHAP waterfall and force plots provided visual explanations of how each variable contributed to a given patient’s predicted probability (Figures 5C,D). For example, in one representative case, a CLR of 30.9 contributed +0.0946 to the risk estimate, Hunt–Hess grade III–IV added +0.161, PCT level of 1.01 added +0.177, and a GCS score of 8–12 added +0.101. The cumulative SHAP values yielded the final model output, offering an interpretable, traceable rationale for each prediction.

By incorporating SHAP, the neural network model provided not only accurate risk estimation but also clinically meaningful justification for its outputs. This enhanced interpretability addresses the “black-box” nature often associated with machine learning and improves the model’s potential for real-world adoption in neurocritical care.

## 4 Discussion

In this study, we developed a risk scoring system for predicting DCI in patients with aSAH by integrating clinical grading scales with routinely available inflammatory biomarkers. Based on a single-center retrospective cohort, we applied Boruta-based feature selection and constructed six supervised machine learning models. The proposed system was independently associated with both DCI occurrence and poor outcomes. Incorporating inflammatory markers into the model significantly enhanced its discriminative performance, supporting its potential utility in individualized risk stratification and clinical decision-making. Our findings may find a potential interrelationship among systemic inflammation, DCI, and neurological outcomes; however, confirming this hypothesis will require prospective studies incorporating formal mediation analyses to elucidate the underlying causal pathways and mechanisms.

Systemic inflammation plays a central role in the pathogenesis of intracranial aneurysms and their complications (16–18). Blood-derived inflammatory indices have become widely used due to their accessibility and scalability (19, 20). However, individual markers have limited sensitivity and are often collinear. To address this, we employed the Boruta algorithm, which effectively reduces redundancy by identifying variables with true predictive value while minimizing the impact of multicollinearity (21, 22). The final model integrated neurologic function (GCS), hemorrhage severity (modified Fisher score), and clinical status (Hunt–Hess grade), alongside immune-nutritional indicators such as PNI, NAR, CLR, and NLPR. This multidimensional strategy reflects the early physiological state more comprehensively than any single parameter. Traditional scores such as GCS and Hunt–Hess remain highly informative: in our model, Hunt–Hess grade ranked highest in SHAP importance, reaffirming the continuing value of bedside assessments in the era of machine learning (23–26). The inclusion of inflammatory biomarkers further improved model performance. Lower PNI reflects impaired nutritional and immune reserve (27), while NAR and CLR quantify the imbalance between inflammation and immune suppression (28, 29). NLPR integrates inflammatory and coagulation pathways and has been validated in stroke and critical care populations (30, 31). Among all features, procalcitonin (PCT) showed the highest SHAP contribution (+0.177), underscoring its value in identifying high-inflammatory phenotypes. Although initially developed for infection monitoring, elevated PCT is now linked to secondary brain injury and poor stroke outcomes (32–35), supporting its role in neurocritical risk assessment.

Among all algorithms tested, the neural network achieved the best balance between discrimination and calibration, with an AUC of 0.826 and Brier score of 0.157 in the training cohort, and stable performance in internal validation. SHapley Additive Explanations (SHAP) provided interpretable insights at both global and individual levels. Key contributors—particularly Hunt–Hess grade and PCT—were visualized using summary, waterfall, and force plots. These tools enhanced model transparency and clinical trust, supporting translation into practice. Overall, this study offers three major contributions: (1) construction of a practical risk score combining clinical and inflammatory data, (2) application of Boruta for robust feature selection, and (3) identification of a neural network as the most stable algorithm with strong interpretability. From a methodological perspective, the exclusion of patients who died during hospitalization helped reduce heterogeneity introduced by early catastrophic neurological deterioration. Such patients often experience rapid clinical collapse that prevents standardized neurological assessment or completion of routine laboratory testing. Including these extreme physiological outliers could have distorted model training and obscured the discriminative contributions of inflammatory biomarkers. Therefore, restricting the analysis to hospitalized survivors allowed for more consistent data acquisition and improved internal validity when evaluating early predictors of DCI.

This study has several limitations. First, it was based on retrospective data from a single center, which may restrict the generalizability of our findings. Furthermore, because patients who died during hospitalization were excluded, the study cohort predominantly represents aSAH survivors with relatively better prognoses. This selection may introduce bias and could lead to an overestimation of model performance when applied to more critically ill populations, particularly those with early fatal deterioration and distinct inflammatory trajectories. Future studies should include more comprehensive cohorts incorporating early mortality cases to enhance external validity and ensure broader applicability. Second, although key treatment-related variables such as endovascular therapy and surgical clipping were included in the analysis, other important therapeutic factors—such as nimodipine administration strategies, blood pressure and fluid management protocols, and the intensity of postoperative monitoring—were not comprehensively captured. Because DCI is highly sensitive to these clinical management details, incomplete adjustment for such variables may introduce residual confounding. In addition, the inflammatory response is a dynamic and time-dependent process, whereas our model relied on laboratory measurements obtained at a single time point on admission, limiting the ability to reflect temporal changes in inflammatory status. Therefore, future prospective, multicenter studies with standardized treatment documentation and longitudinal biomarker monitoring are needed to validate the model’s performance and further clarify the influence of dynamic inflammatory trajectories on DCI prediction.

## 5 Conclusion

In conclusion, we developed and internally validated a machine learning–based risk prediction model for delayed cerebral ischemia after aneurysmal subarachnoid hemorrhage, integrating clinical grading scales with peripheral inflammatory biomarkers. The final model, constructed using a neural network algorithm and interpreted via SHAP, demonstrated robust discriminative performance and clinical interpretability. This practical and accessible scoring system may aid early identification of high-risk patients and support individualized management strategies in neurocritical care. Prospective multicenter studies are warranted to confirm generalizability and assess their potential for clinical integration.

## Data Availability

The original contributions presented in the study are included in the article/Supplementary material, further inquiries can be directed to the corresponding author.
