# Supplementary material for: Machine learning–driven risk prediction of delayed cerebral ischemia after aneurysmal subarachnoid hemorrhage using peripheral inflammatory markers
Source: Front Neurol. 2025 Dec 11;16:1713341. doi: 10.3389/fneur.2025.1713341 (PMC12738297; doi:10.3389/fneur.2025.1713341)
Supplement: Supplementary file 1 [file Supplementary_file_1.docx]

**Supplementary File 1: Parameters of the six machine learning algorithms**

#Model training

models = c("glm","svmRadial","gbm","nnet","extraTrees","xgbTree")#

#Model name

models_names = list(Logistic="glm",SVM="svmRadial",GBM="gbm",NeuralNetwork="nnet",RandomForest="extraTrees",Xgboost="xgbTree")#

#Parameter settings

glm.tune.grid = NULL

svm.tune.grid = expand.grid(sigma = 0.001, C = 0.09)

gbm.tune.grid = expand.grid(n.trees = 100, interaction.depth = 5,shrinkage = 0.1, n.minobsinnode = 30)

nnet.tune.grid = expand.grid(size = 6,decay = 0.6)

rf.tune.grid = expand.grid(mtry = 11,numRandomCuts = 3)

xgb.tune.grid = expand.grid(nrounds = 10,max_depth = 3,eta = 0.001,

gamma = 0.5,colsample_bytree = 0.5,min_child_weight = 1,subsample = 0.6)

Tune_table = list(glm = glm.tune.grid,

svmRadial = svm.tune.grid,

gbm = gbm.tune.grid,

nnet = nnet.tune.grid,

extraTrees = rf.tune.grid,

xgbTree = xgb.tune.grid

)

#Model

ML_calss_model = list()

set.seed(52)

train.control <- trainControl(method = 'repeatedcv',

number = 10,

repeats = 5,

classProbs = TRUE,

summaryFunction = twoClassSummary)
